# Monolithic and Single-Crystalline Aluminum–Silicon Heterostructures

**DOI:** 10.1021/acsami.2c04599

**Published:** 2022-05-27

**Authors:** Lukas Wind, Raphael Böckle, Masiar Sistani, Peter Schweizer, Xavier Maeder, Johann Michler, Corban G.E. Murphey, James Cahoon, Walter M. Weber

**Affiliations:** † Institute of Solid State Electronics, Technische Universität Wien, Gußhausstraße 25-25a, 1040 Vienna, Austria; ‡ Swiss Federal Laboratories for Materials Science and Technology, Laboratory for Mechanics of Materials and Nanostructures, Feuerwerkstrasse 39, 3602 Thun, Switzerland; § Department of Chemistry, University of North Carolina, Chapel Hill, North Carolina 27599-3290, United States

**Keywords:** silicon, aluminum, metal−semiconductor heterostructure, Schottky barrier field effect transistor, solid-state reaction

## Abstract

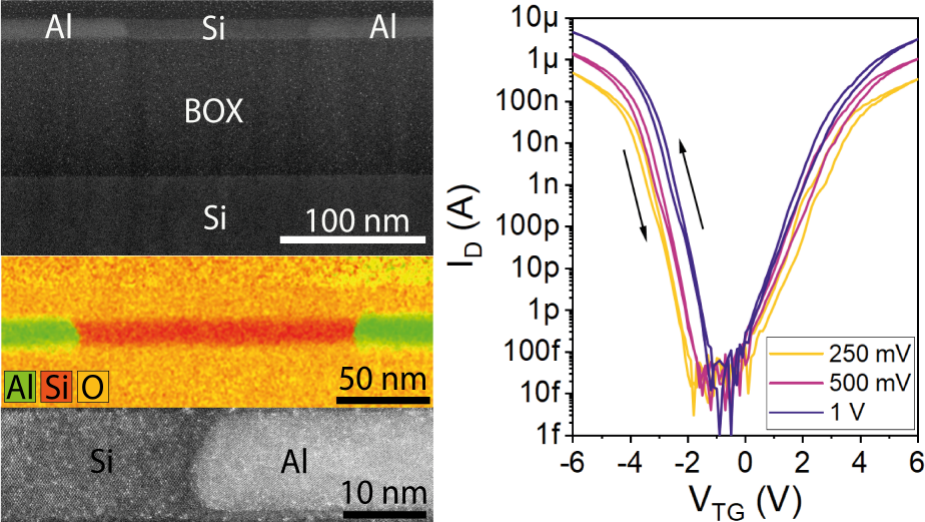

Overcoming the difficulty in the precise definition of the metal phase of metal–Si heterostructures is among the key prerequisites to enable reproducible next-generation nanoelectronic, optoelectronic, and quantum devices. Here, we report on the formation of monolithic Al–Si heterostructures obtained from both bottom-up and top-down fabricated Si nanostructures and Al contacts. This is enabled by a thermally induced Al–Si exchange reaction, which forms abrupt and void-free metal–semiconductor interfaces in contrast to their bulk counterparts. The selective and controllable transformation of Si NWs into Al provides a nanodevice fabrication platform with high-quality monolithic and single-crystalline Al contacts, revealing resistivities as low as ρ = (6.31 ± 1.17) × 10^–8^ Ω m and breakdown current densities of J_*max*_ = (1 ± 0.13) × 10^12^ Ω m^–2^. Combining transmission electron microscopy and energy-dispersive X-ray spectroscopy confirmed the composition as well as the crystalline nature of the presented Al–Si–Al heterostructures, with no intermetallic phases formed during the exchange process in contrast to state-of-the-art metal silicides. The thereof formed single-element Al contacts explain the robustness and reproducibility of the junctions. Detailed and systematic electrical characterizations carried out on back- and top-gated heterostructure devices revealed symmetric effective Schottky barriers for electrons and holes. Most importantly, fulfilling compatibility with modern complementary metal–oxide semiconductor fabrication, the proposed thermally induced Al–Si exchange reaction may give rise to the development of next-generation reconfigurable electronics relying on reproducible nanojunctions.

## Introduction

The rapid advancement of the miniaturization of microelectronic components aided the development of paradigms and devices enabling information technologies, which are omnipresent in our everyday life.^1^ However, the ever-shrinking feature sizes of Si metal–oxide semiconductor field effect transistors (MOSFETs) leads to fundamental scaling limits as increased leakage currents and relatively high supply voltages, which restrict enhancing the performance of modern devices.^2,3^ Further, the increased complexity of integrated circuits results in an ever-growing power consumption due to parasitic capacitances and resistances of interconnect lines.^4^ In parallel to this development, rising computing paradigms such as the “Internet of Things” and “artificial intelligence” are demanding the design of systems with even higher computational resources. In this regard, the functional diversification of transistors constitutes alternative approaches to enable novel system concepts enhancing state-of-the-art solutions.^5,6^ To overcome the scaling limitation and therefore enhance novel device concepts, it is mandatory to implement new processes and device architectures to enable “more-than-Moore” paradigms^7^ extending the mature Si complementary metal–oxide semiconductor (CMOS) platform. A major prerequisite for a large number of emerging nanoelectronic, optoelectronic, and quantum devices are reliable and reproducible metal–semiconductor junctions. A possibility to enable such contacts is exchanging the semiconductor with metal by thermally induced diffusion processes. Thus, thorough research on the thermal diffusion of metals in Si and Ge to form silicide^8,9^ and germanide^10^ metallic compound materials has been carried out.^11^ Importantly, material combinations with no intermetallic phase formation can overcome difficulties in the precise and reproducible definition the crystal phase and stoichiometry of the intruded metallic segments. These systems enable single-elementary metal–semiconductor heterostructures and have received strong attention.^12−18^ However, up to now, monolithic heterostructures between elementary metal and Si are still elusive, which is mainly attributed to either stress-induced voiding and void nucleation via electromigration^19−22^ or interdiffusion, causing unwanted doping.^23−25^

## Results and Discussion

**Figure 1:**
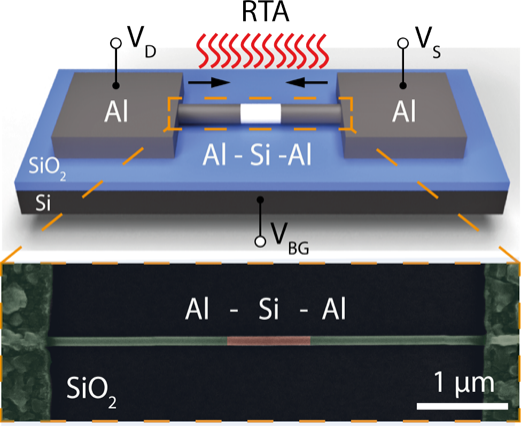
Schematic illustration of the Al–Si–Al NW heterostructure. The highly p-doped Si wafer is used as global back-gate. The false-color SEM image shows a device with a Si channel length of *L*_Si_ = 1 μm.

In this paper, we report on a CMOS compatible technology to form monolithically integrated Al–Si heterostructures in the nanometer scale exhibiting geometrically abrupt metal–semiconductor interfaces. The presented approach relies on a thermally induced exchange reaction between Al and Si and was investigated for completeness on both bottom-up grown Si nanowires (NWs) and top-down fabricated Si nanosheets patterned from silicon-on-insulator (SOI) wafers. To assess the capabilities of this approach and material system, we systematically investigated the electrical properties of Al–Si heterostructures by considering Schottky barrier field effect transistors (SBFET). To investigate the Al–Si exchange, vapor–liquid solid^26^ (VLS) grown ⟨111⟩-oriented Si NWs wrapped in a 10 nm thermally grown SiO_2_ shell were transferred on a highly p-doped Si substrate with a 100 nm thick SiO_2_ and contacted by Al pads (see Figure 1). For monolithic contact formation, a thermally induced exchange reaction between the Si NWs and Al contact pads was employed using rapid thermal annealing (RTA) at *T* = 774 K. The false-color scanning electron microscopy (SEM) image in Figure 1 shows bright segments (colored in green) emerging from the lithographically defined Al contact pads, which for prolonged annealing extend within the Si NW (colored in red). The formation mechanism of the Al–Si NW heterostructure can be best elucidated by analyzing both the Al–Si phase diagram (see Figure S1)^27^ and considering the strong asymmetric diffusion kinetics of the Al–Si material system (see Table 1).^28,29^ Importantly, the diffusion of Si in Al as well as the Al self-diffusion (i.e., Al in Al) is comparatively high, whereas the diffusion of Al in Si is 14 and 12 orders of magnitude smaller, respectively. Thus, Al atoms are supplied via a highly efficient self-diffusion mechanism through the already-formed Al segment and finally transported to the interface to the pristine Si NW segment, where they compensate the out-diffusion of Si atoms. Further, based on the diffusion coefficients and assuming that Si diffusion in Al takes place through interstitials,^22^ it is assumed that Si atoms can diffuse across the entire exchanged Al segment and ultimately through the Al pads and/or to the structure surface depending on the available surface passivation. The bulk binary Al–Si phase diagram has a single eutectic point and shows no solid intermetallic stoichiometry. The melting points of Al and Si are at *T* = 993 and 1684 K, respectively. As the eutectic temperature of the Al–Si system is located at a composition of approximately 12.6 wt % Si, we assume that the reported exchange process performed at *T* = 774 K occurs as a solid-state reaction. Indeed, the solubility of Al in solid Si is reported to be very low.^30^ Considering this solubility gap and the largely asymmetric diffusion coefficients, we assume that there is effectively no electrically active Al within the Si segment. Investigations of the Al–Si exchange in ⟨112⟩-oriented Si NWs contacted by Al pads revealed a rate of (25.9 ± 2.3) nm s^–1^ at *T* = 774 K. We attribute this variation to different Al–Si contact surfaces, i.e., residual patchy oxide layers on the contact area between the Si NW and the Al pads, which might cause different exchange rates. Importantly, the presented Al–Si exchange allows to perform consecutive annealing cycles to define the Si channel monolithically connected to the Al leads. This constitutes a significant advantage over state-of-the-art silicide formation processes, which encounter phase changes upon further annealing.^8^

**Table 1:** Calculated Diffusion Coefficients of the Al–Si Material System for a Temperature of *T* = 774 K^28,29^

Al in Al (cm^2^ s^–1^)	Al in Si (cm^2^ s^–1^)	Si in Al (cm^2^ s^–1^)	Si in Si (cm^2^ s^–1^)
6.3 × 10^–10^	2.0 × 10^–22^	4.4 × 10^–8^	6.5 × 10^–19^

For extended annealing, the NW appeared to be composed of pure Al with a resistivity of ρ = (6.31 ± 1.17) × 10^–8^ Ω m, which is less than three times larger than that of bulk Al^31^ and can be attributed to a size effect given by the increased surface scattering in NWs compared to bulk.^32^Figure S2 shows the resistivity of such Al NWs obtained from two-point *I*/*V* measurements in the temperature range between *T* = 77.5 and 400 K. In agreement with the decrease of phonon scattering at lower temperatures of metals,^33^ a decreasing resistivity of such Al NWs was found. Importantly, as the resistivity of the obtained Al NWs is approximately five orders of magnitude smaller compared to the used Si NWs, which have a resistivity of ρ = (2.2 ± 1.2) × 10^3^ Ω m, the parasitic resistance of the Al leads to the Si channel should be negligible. Further, remarkably high breakdown current densities of *J*_max_ = (1 ± 0.13) × 10^12^ Ω m^–2^, comparable with Ni*_x_Si*_1 – *x*_–Si NWs were obtained.^34^ Moreover, below the transition temperature of Al (*T_c_* = 1.25 K),^35^ our Al–Si–Al heterostructures could be a highly interesting building block for superconductor–semiconductor hybrid devices such as, e.g., gate-tunable Josephson junctions and superconducting qubits.^36^ Using other common material systems such as binary nickel silicides this has not been possible due to Ni being ferromagnetic, which inhibits superconductivity.

**Figure 2:**
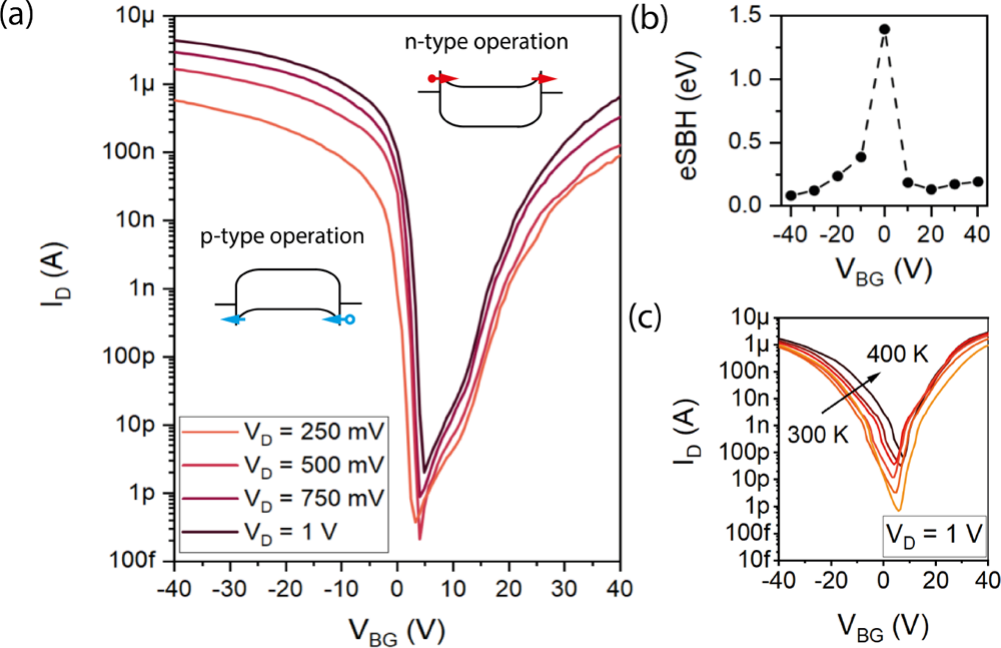
(a) Transfer characteristic of an Al–Si–Al NW heterostructure device with a Si channel length of *L*_Si_ = 1 μm for *V*_D_ between 250 mV and 1 V. The arrows indicate the gate voltage sweeping direction. The band diagrams for hole and electron conduction are inserted. (b) Effective Schottky barrier as a function of *V*_BG_. (c) Temperature-dependent transfer characteristic for *V*_D_ = 1 V.

To assess the applicability of our novel junctions in the realization of nanoelectronic devices, the Al–Si–Al NW heterostructures were first operated as back-gated NW FETs using the p-doped Si substrate as a common back-gate to determine their modulation capabilities. Figure 2a shows the typical transfer characteristic of such a back-gated NW FET device with a length of *L*_Si_ = 1μm and a diameter of *d*_NW_ = 80 nm. Applying drain bias voltages between *V*_D_ = 250 mV and 1 V, the device shows an *I*_On_/*I*_Off_ ratio of 10^8^ and exhibits a pronounced ambipolar transfer characteristic with predominant hole transport for *V*_BG_ < 5 V (p-type operation) and predominant electron transport for *V*_BG_ > 5 V (n-type operation). The characteristics imply the presence of Schottky barriers both for the injection to the conduction band and valence band edges. Assuming thermionic emission, an effective Schottky barrier height (eSBH) for electrons and holes was obtained in dependence of the applied gate voltage from the slope of the Arrhenius activation energy representation of ln(*J*/*T*^2^) vs 1000/*T* (see the Supporting Information). As shown in Figure 2b, relatively symmetric barriers for holes and electrons of *q*ϕ_eSBH_ = 82 meV (*V*_BG_ = −40 V) and 119 meV (*V*_BG_ = 40 V) were obtained. So far, it has been difficult to find symmetric barrier heights for Schottky junctions to Si with metals or even metal silicides;^37,38^ these, however, are expected to enhance the behavior of reconfigurable transistors.^39−43^ Further, the current density for holes *J*_h_ equals to 9 × 10^8^ A m^–2^ and, for electrons, *J*_e_ = 1 × 10^8^ A m^–2^. The off-current density for the back-gated NW device is *J*_Off_ = 397 A m^–2^. This is in contrast to other monolithic metal–semiconductor heterojunctions such as the Al–Ge system, which reveals a highly transparent contact for holes and a pronounced barrier for electrons.^6,44^Figure 2c shows the effect of temperature on the transfer characteristic for *V*_D_ = 1 V between *T* = 300 and 400 K. As can be seen, a gate voltage shift and an increase of the off-current with temperature are evident, which can be attributed to the injection of thermally generated carriers over the Schottky barrier. However, no substantial increase of the on-current was observed, which is in agreement with a tunneling-dominated charge injection.

**Figure 3:**
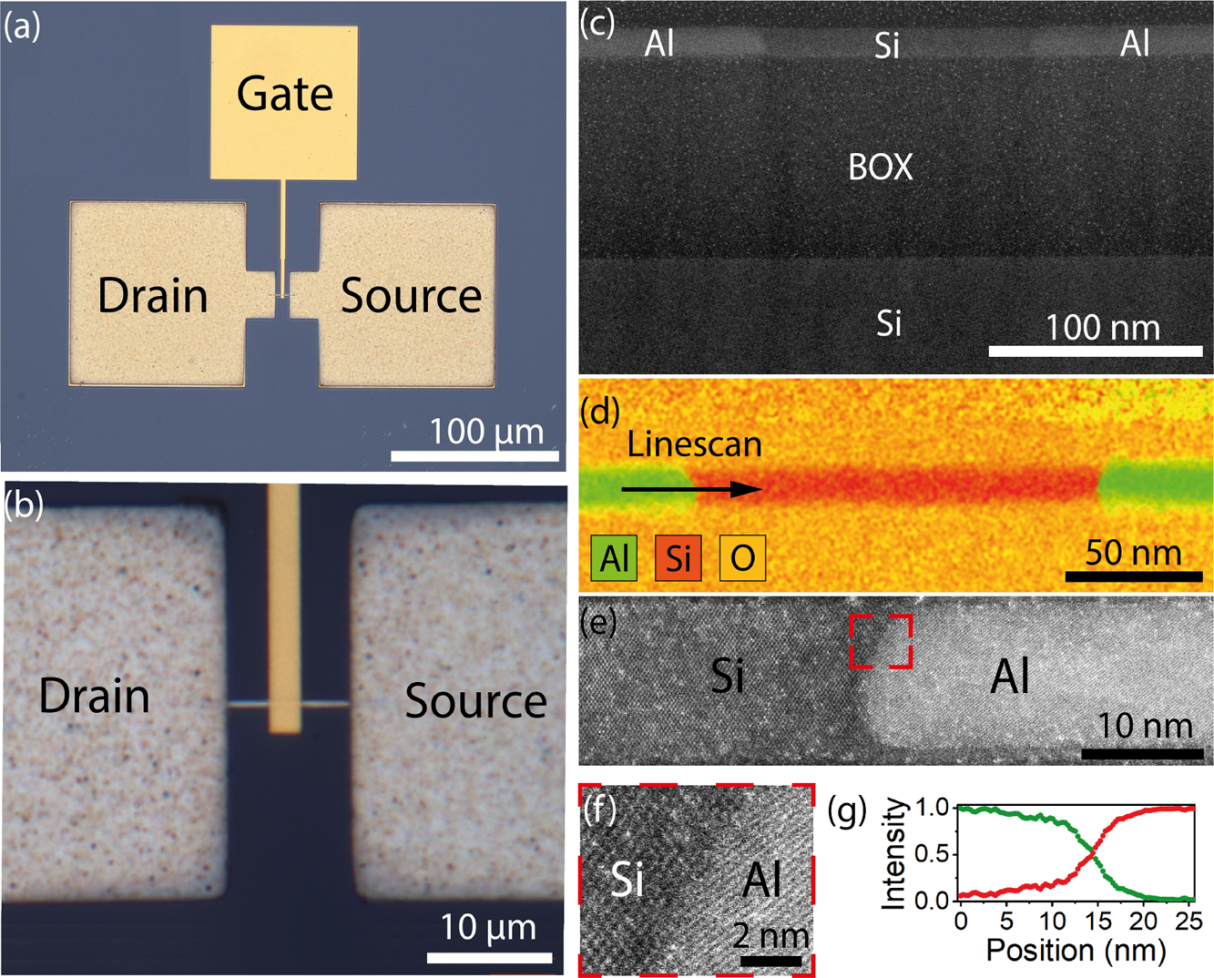
(a) Overview and (b) close-up microscope image of a top-down fabricated top-gated heterostructure. (c) TEM image of a top-down fabricated Al–Si–Al device and respective EDX map of the heterostructure (d). HRTEM image showing an Al–Si interface (e) and a close-up image at the red dashed box shown in (f). An EDX linescan across the abrupt Al–Si junction as indicated in (d) is shown in (g).

To demonstrate the impact of Al–Si–Al heterostructures on top-down fabricated nanosheets and to improve the electrostatic control of the Si channel, an omega-shaped top-gate was fabricated atop top-down fabricated nanosheets with a thickness of 15 nm enwrapped in a 12 nm SiO_2_ shell based on fully depleted SOI substrates. Figure 3a,b shows an overview and close-up microscope image of a top-down fabricated top-gated Al–Si–Al heterostructure device with source/drain and an overlapping top-gate contact. To investigate the Al–Si interface and the elemental composition of the structure in more detail, structural analysis based on transmission electron microscopy (TEM) and energy-dispersive X-ray spectroscopy (EDX) were performed. Figure 3c shows a cross-sectional TEM image along the entire Al–Si–Al heterostructure on 100 nm thick buried SiO_2_ (BOX) and the Si substrate underneath. Along the entire heterostructure, the TEM analysis of the investigated sample did not reveal any signs of void formation in the bulk Al contacts due to the Al–Si exchange. The EDX measurement in Figure 3d, with a linescan along the abrupt Al–Si interface in Figure 3g, indicates a complete replacement of the Si by the Al during the thermal exchange reaction. Remarkably, no Al contamination in the remaining Si segment was detected within the resolution limit of the EDX (<1%). A cross-sectional high-resolution (HR) TEM image in Figure 3c shows an abrupt Al–Si interface junction. In contrast to bulk Al–Si junctions, the nanoscale junctions show the absence of voids and contact spiking features as well as the absence of intermetallic phases after the exchange process. Importantly, the crystal phase stability of the proposed Al–Si contact formation overcomes the difficulty with the complex growth kinetics of common Ni_*x*_Si_1–*x*_–Si heterostructures, which exhibit strong variability and yield issues.^45^ The enlarged view of the HRTEM image (Figure 3e) in Figure 3f shows the differences of the lattice constants and crystal orientations of the formed crystalline Al lead and the remaining Si segment at the abrupt interface. Local fast Fourier transform (FFT) patterns of the Al and Si region are shown in Figure S3. While the remaining Si segment showed a diamond structure, the Al part of the heterostructure was identified as a face-centered cubic structure. Both crystals are oriented in a [110] zone axis with a mutual in-plane rotation to each other. This rotation presumably leads to the reduction of mechanical strain to accommodate the lattice mismatch between Al and Si. Analyzing the Al–Si exchange process for nanosheets with different widths between *W* = 300 and 700 nm (see Figure S4), it is evident that the exchange rate increases for narrower geometries, showing a 1/ dependency as in Ni–silicide nanowire reactions,^46^ which indicates a surface-limited Al–Si exchange.^17^ Compared to the silicidation rates of Ni_*x*_Si_1–*x*_–Si NW heterostructures,^47^ the Al–Si exchange showed less variations, which might indicate a stable crystal phase of the intruded metallic segments. Finally, we want to highlight the differences of our Al–Si exchange reaction to the already known metal–silicide solid-state reactions in Si NWs and nanosheets (e.g., NiSi,^45,46,48^ PtSi,^49^ CoSi,^50^ and PdSi^51^). In these material systems, the diffusing metal atoms react with the host Si lattice and form a compound phase at the interface to the pristine Si. Consequently, Si diffusion only plays a minor role in the metal silicide solid-state reactions listed above, e.g., in Ni–disilicide, most Si is retained as the NiSi_2_ structure allows for a comparable Si density to the Si host lattice. Distinctly different to this process, in our Al–Si system, we do not find the remaining Si within the intruded and reacted region. Supported by the paramount difference in diffusion coefficients (Table 1), we propose that Si is out-diffused and fully replaced by Al. The thereof obtained single-elementary Al–Si heterostructures not only are highly interesting for nanoelectronic and quantum electronic applications but also provide vast opportunities for near-infrared plasmon enhanced optoelectronic devices,^36^ in particular, plasmon-assisted photodetectors^52^ and monolithic plasmon detectors^53^ comprising plasmonic waveguides (Al contacts) with an attached detector (Si channel).^54^

**Figure 4:**
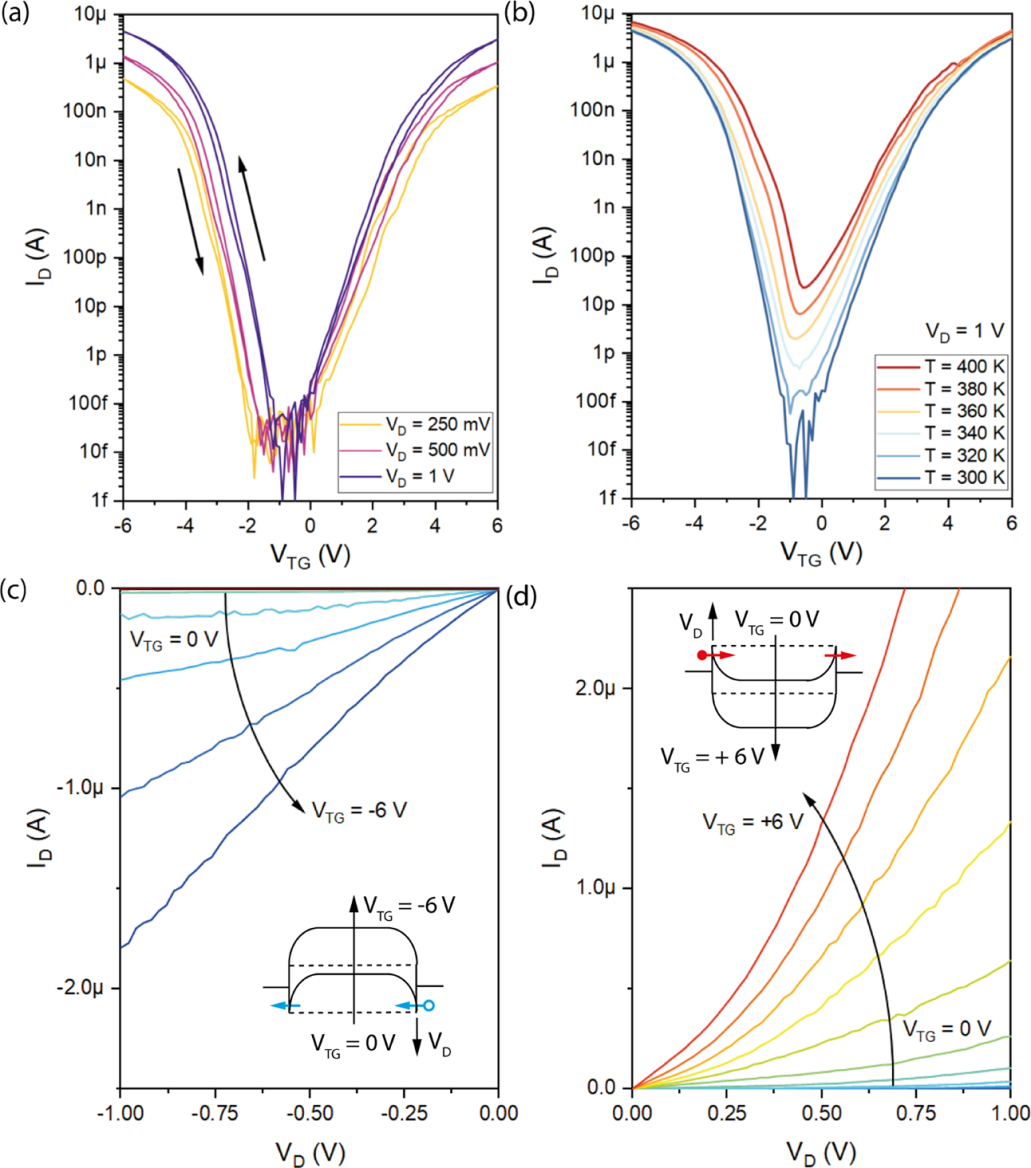
(a) Transfer characteristic of a top-down fabricated Al–Si–Al device with *L*_Si_ = 1**μ**m for *V*_D_ between 250 mV and 1 V. (b) Temperature dependent transfer characteristic for *V*_D_ = 1 V. Linear output characteristic of the (c) hole and (d) electron conduction. The respective band diagrams are inserted accordingly.

Figure 4 discusses the electrical characteristics of top-gates fabricated on top-down Al–Si–Al heterostructures operated as SBFETs. In this respect, Figure 4a shows the transfer characteristics of a device with *L*_Si_ = 1 μm, *W* = 430 nm, and *H* = 15 nm for a drain bias voltage between *V*_D_*=* 250 mV and 1 V. A pronounced and symmetric ambipolar characteristic with hole-driven transport for *V*_TG_ of <−1 V and electron-driven transport for a *V*_TG_ of >0 V is observed. While a pronounced hysteresis is evident due to absorbates on the Si channel exposed at ambient air and the capacity influence of the back-gate (see Figure S5), top-gated devices show minimal dependency on the gate voltage sweeping direction. An *I*_On_/*I*_Off_ ratio of up to 10^8^ for both hole and electron conduction is observed for a bias voltage of *V*_D_ = 1 V. Further, relatively symmetric peak current densities of the p-mode *J*_h_ = 7 × 10^8^ A m^–2^ and n-mode *J*_e_ = 4.8 × 10^8^ A m^–2^ are obtained. The off-current density of the single top-gate device is *J*_Off_ ≈ 1 A m^–2^. Remarkably, for the back-gated NW devices similar current densities were extracted. For an appropriate comparison, the quasi-diameter *d*_NS_ of the nanosheets needs to be considered. Therefore, *A*_NS_ ≔ *A*_NW_ needs to be fulfilled, leading to a quasi-diameter of *d*_NS_ = . Applying this relation the equivalent nanosheet diameter can be calculated with *d*_NS_ = 89.5 nm, which is comparable to the NW diameter *d*_NW_ of 80 nm. Figure 4b depicts the temperature evolution of the subthreshold transfer characteristic for V_*D*_ = 1 V from *T* = 300 to 400 K, which is also significantly improved. While the back-gated device showed a severe shift of the intrinsic point in the investigated temperature range, top-gated devices reveal only a thermally induced increase of the off-current for increasing temperature. The low off-current of the Al–Si–Al heterostructures in combination with the high-temperature sensitivity in this region would be the preferred system for active Si bolometers.^55^ Importantly, the symmetric ambipolar device operation remains stable in the investigated temperature range. Further, the output characteristics for hole and electron conduction as well as their respective schematic band diagrams are shown in Figure 4c,d, respectively. Typical for SBFETs,^56,57^ both output characteristics reveal a supralinear behavior for a small *V*_D_, with the on-currents showing a high variation.

## Conclusions

In conclusion, we have systematically investigated Al–Si–Al heterostructures based on both bottom-up grown Si NWs and top-down-fabricated nanosheets. This was achieved via a thermally induced Al–Si exchange reaction, enabling the monolithic integration of Si channels with high-quality Al contacts and revealing resistivities as low as ρ = (6.31 ± 1.17) × 10^–8^ Ω m and breakdown current densities of *J*_max_ = (1 ± 0.13) × 10^12^ Ω m^–2^. Importantly, the obtained Al–Si junctions revealed symmetric eSBHs for holes and electrons, which is an important prerequisite for reconfigurable electronics. The structural analysis by TEM and EDX confirmed the high quality and abruptness of the obtained Al–Si junctions. Importantly, the crystal phase stability of the proposed Al–Si contact formation overcomes the difficulty with the complex growth kinetics of common silicide–Si contacts, resulting in strong variability and yield issues. Embedded in back- and top-gate SBFET architectures, the electrical transport properties of the proposed Al–Si–Al heterojunctions were systematically probed and investigated. Integrated into a top-gated SBFET architecture and applying a bias voltage of *V*_D_ = 1 V, we calculated relatively symmetric peak current densities of the p-mode *J*_h_ = 7 × 10^8^ A m^–2^ and the n-mode *J*_e_ = 4.8 × 10^8^ A m^–2^. Remarkably, an *I*_On_/*I*_Off_ ratio of 10^8^ was obtained. Most notably, enabling the wafer-scale accessibility of high-quality Al–Si–Al heterostructures, the proposed architecture may pave the way for emerging SBFET devices for next-generation reconfigurable electronics based on monolithic metal–semiconductor heterostructures.

## Methods

### Bottom-Up Device Fabrication

The ⟨112⟩ oriented Si NWs were grown in a hot-wall chemical vapor deposition (CVD) system using silane (SiH_4_, Voltaix), hydrogen chloride (HCl anhydrous; Matheson TriGas, 5N research purity grade), and hydrogen (H_2_, Matheson TriGas, 5N semiconductor grade) as the carrier gas, following protocols similar to those reported previously.^58^ Au nanoparticle catalysts of diameter 80 nm (Sigma-Aldrich) were immobilized on growth substrates composed of Si wafers with 600 nm thermal oxide (NOVA Electronic Materials) by functionalization of the substrate with poly-l-lysine (Sigma-Aldrich) followed by functionalization with Au nanoparticles. Substrates were cleaned in a UV-ozone cleaner and inserted in a 1 inch quartz-tube furnace (Lindberg Blue M) for growth by CVD. NW growth was nucleated at 753 K with 2 standard cubic centimeters per minute (sccm) of SiH_4_, 4 sccm of HCl, and 194 sccm of H_2_ at 20 torr total reactor pressure for 20 min, and these conditions were maintained for an additional 80 min to reach the desired NW length of 20 μm. Subsequent to the growth, the Si NWs were thermally oxidized at *T* = 1174 K in O_2_ atmosphere for 3 min and annealed for 3 min in N_2_ atmosphere to employ a high-quality SiO_2_ gate-oxide. Subsequently, the Si NWs were transferred onto a highly p-doped oxidized Si wafer. Al contacts to the Si NWs were fabricated by a combination of electron beam lithography, 15 s of BHF (7:1) etching to remove the SiO_2_-shell at the contact area, 125 nm Al sputter deposition, and lift-off techniques. RTA at a temperature of *T* = 774 K in forming gas atmosphere was employed to initiate an Al–Si exchange.

### Top-Down Device Fabrication

The devices were fabricated from SOI substrates composed of a 20 nm thick (100) oriented Si device layer on top of a 100 nm thick BOX and a 500 μm thick and lowly doped Si substrate. The patterning of the Si structures was done by laser lithography and SF_6_–O_2_ based reactive ion etching. Thermal oxidation at *T* = 1174 K in O_2_ atmosphere for 10 min and an annealing procedure for 10 min in N_2_ atmosphere were employed to fabricate a high-quality SiO_2_ gate-oxide. Al pads contacting the Si nanostructures were fabricated by laser lithography, 125 nm Al sputter deposition preceded by a 15 s BHF dip (7:1) to remove the SiO_2_ at the contact area, and lift-off techniques. The Al–Si exchange reaction was induced by rapid thermal annealing at a temperature of *T* = 774 K in forming gas atmosphere. An omega-shaped top-gate architecture was realized using laser lithography, Ti/Au evaporation (10 nm Ti and 100 nm Au), and lift-off techniques.

### TEM Imaging

The TEM lamella was fabricated by employing a dual beam FIB/SEM tool (model: Tescan Lyra). The lamella was lifted and transferred to a TEM (model: Thermo Fisher Scientific Titan Themis 200 G3) complemented with a SuperX detector for EDX mapping.

### Electrical Measurements

The electrical measurements took place employing a semiconductor parameter analyzer (HP 4156B) and a shielded and dark probe station. Temperature-dependent measurements as well as measurements to extract the effective Schottky barrier heights were performed in a vacuum using a cryogenic probe station (model: LakeShore PS-100) and a semiconductor parameter analyzer (model: Keysight B1500A).

## Supporting Information Available

Al–Si phase diagram; temperature-dependent resistivity of Al NWs obtained from Al–Si exchange; HRTEM image of the Al–Si interface with indexed FFT patterns of the Si and Al segments; investigations of the Al–Si exchange rate versus the nanosheet width of the Si channel; hysteresis of the transfer characteristic of a back-gated Al–Si–Al NW heterostructure depending on the voltage sweep direction; detailed discussion of the performed gate-dependent *q*ϕ_eSBH_ extraction (PDF)
