# Supplementary material for: Monolithic and Single-Crystalline Aluminum–Silicon Heterostructures
Source: ACS Appl Mater Interfaces. 2022 May 27;14(22):26238–44. doi: 10.1021/acsami.2c04599 (PMC9185687; doi:10.1021/acsami.2c04599)
Supplement: Supplementary file 1 — am2c04599_si_001.pdf [file am2c04599_si_001.pdf]

Supporting Information: Monolithic and Single-Crystalline Aluminum-Silicon Heterostructures

Lukas Wind,[†,§] Raphael Böckle,[†,§] Masiar Sistani,[†] Peter Schweizer,[‡] Xavier Maeder,[‡] Johann Michler,[‡] Corban G.E. Murphey,[¶] James Cahoon,[¶] and Walter M. Weber*,[†]

†*Institute of Solid State Electronics, Technische Universität Wien, Gußhausstraße 25-25a, 1040 Vienna, Austria*

‡*Swiss Federal Laboratories for Materials Science and Technology, Laboratory for Mechanics of Materials and Nanostructures, Feuerwerkstrasse 39, 3602 Thun, Switzerland*

¶*Department of Chemistry, University of North Carolina, Chapel Hill, 27599-3290 North Carolina, United States*

§*These authors contributed equally*

E-mail: walter.weber@tuwien.ac.at

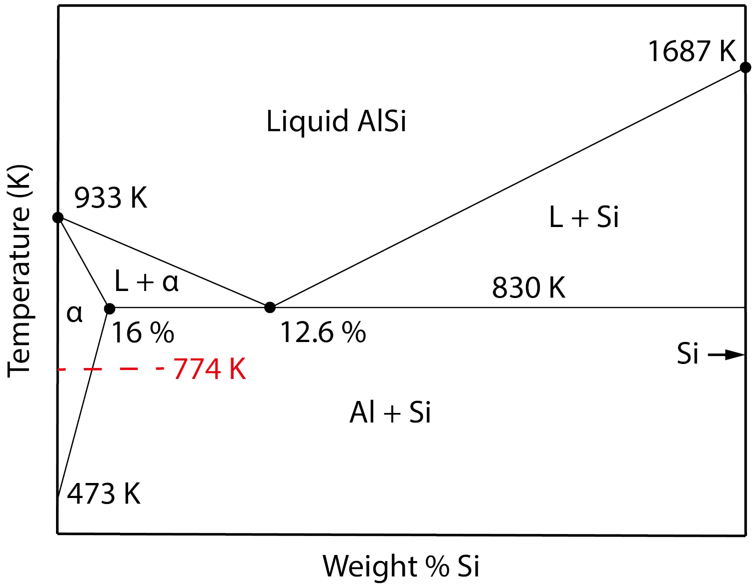

Figure S1: Binary Al-Si phase diagram showing a simple eutectic type with no intermetallic phase formation.[1] The melting points of Al and Si are 993 K and 1684 K, respectively, and the eutectic point is located at composition of about 12.6 wt % Si with a solid to liquid transition at 850 K. The applied annealing temperature of 774 K for thermally induced Al-Si formation is indicated in red.

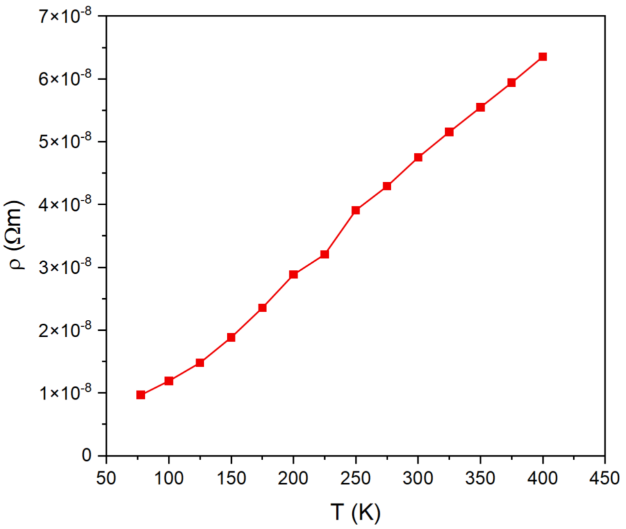

Figure S2: Temperature dependent resistivity of an Al NW between T = 77.5 K and 400 K obtained from thermally induced Al-Si exchange at T = 774 K. Due to the decrease of phonon scattering at lower temperatures in metals,[2] the resistivity is improved for lower temperatures.

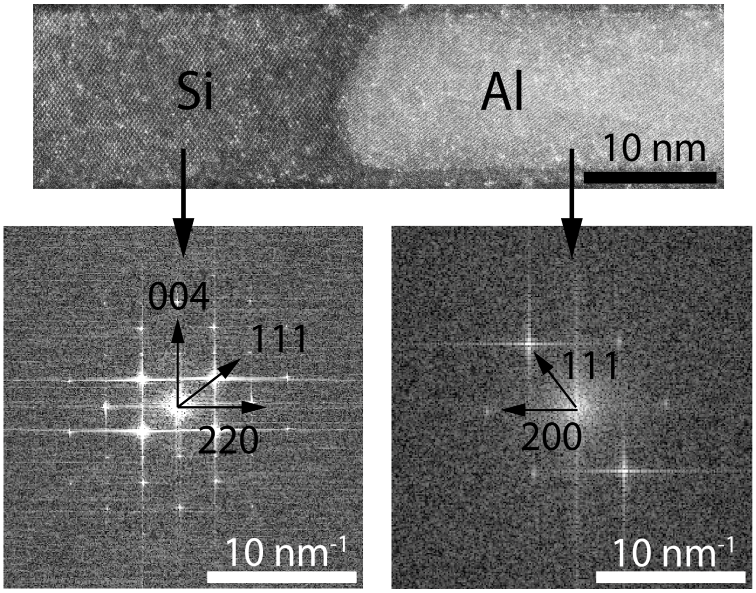

Figure S3: HRTEM image (reproduced from Figure 3e) of the Al-Si interface with indexed FFT patterns (zone axis [110]) of the Si and Al segments.

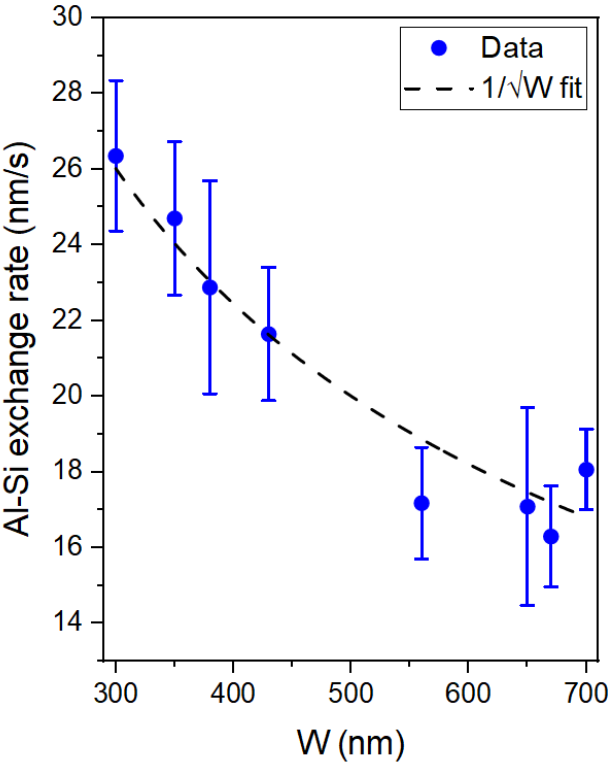

Figure S4: Al-Si exchange rate versus the nanosheet width of the Si channel. The data was fitted using a $1/\sqrt{W}$ function.

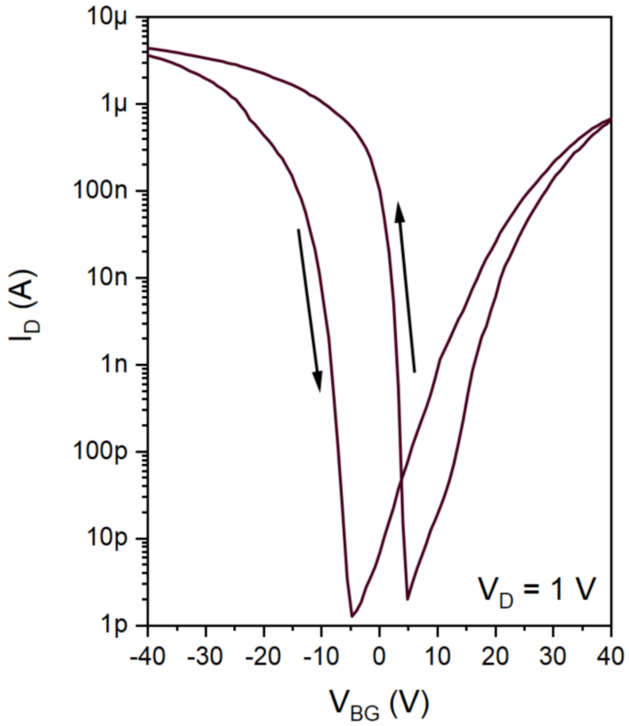

Figure S5: Transfer characteristic of a back-gated Al-Si-Al NW heterostructure with a Si channel length of $L_{Si} = 1\,\mu m$ for $V_D = 1\,V$ showing a pronounced hysteresis depending on the voltage sweep direction (see arrows).

Evaluation of gate-dependent eSBH

The extraction of the eSBH, also denoted as total effective activation energy, is determined for NW- and nanosheet-based Al-Si-Al heterostructures. Analyzing the eSBH allows to investigate the symmetry of the transfer characteristic of the proposed device architectures, and additionally gives an experimental approach to quantitatively describe the injection capability of charge carriers into the Si segment. As symmetric barriers are crucial for the realization of RFETs, the injection and suppression capabilities of charge carriers are important. In consequence, the total effective activation energy is evaluated in dependence of the gate-voltage. For any gated device the transfer characteristic needs to be evaluated prior to the extraction of the activation energy. This allows to identify the intrinsic point as well as dominant n- and p-type operation regimes. As shown in Figure 4b the p- and n-type operation mode reveal similar eSBHs of 130 meV at relatively high V_{BG} for p- and n-type

operation. This is attributed to the fact that the Fermi level of the Al-Si material system seems to pin close to midgap, leading to this symmetric eSBH values.

Experimental eSBH modelling

In the work "Metal-Semiconductor Contacts" by E.H. Rhoderick and R.H. Williams various approaches for the extraction of the Schottky barrier height are discussed.[3] As in this work intrinsic Si is used, the I/V(-T) approach is utilized, which relies on thermionic emission theory. In general, this theory is valid for barrier heights larger than k_BT (25.7 meV at T = 300 K) and small bias voltages to avoid barrier lowering, and thus significant tunneling currents. Moreover, within the standard model, the potential between the metal and semiconductor needs to be taken into account as well. Here, the standard J(T,E) model for Schottky contacts cannot be utilized straight-forward, as the used Al-Si-Al heterostructure does not fully compromise these boundary conditions. Nevertheless, the model can be utilized to get an approximation of the eSBH, which means that the total activation energy for the injection of charge carriers can be evaluated experimentally. The total activation energy includes contributions, which can be attributed to thermionic as well as tunneling emission. Moreover, in the proposed heterostructures two Schottky contacts are involved. In the standard J(T,E) model for, i.e. Schottky diodes, the potential between the metal and doped semiconductor can be directly measured, whereas for SBFETs the electrostatic situation is much more complex because the potential at the semiconductor region cannot be directly determined by static electrical measurements. Different from a Schottky diode, the semiconductor potential depends on the applied gate- and drain-voltages and also on the accumulated charges inside the active region. In addition, the presence of the second Schottky barrier, i.e. at drain makes the calculation of the barrier height more complex, as within a given potential landscape, injection of holes from the drain junction can become considerable. Taking these given boundary conditions into account it is not possible to apply the physically correct expression. Therefore, a rough estimation of the total effective

activation energy of the system can be given. According E.H. Rhoderick and R.H. Williams the current through the Schottky barrier can be simplified in the case that the applied bias voltage exceeds $3 \times k_B T/q$ (76 mV at T = 300 K). Equation 1 gives the simplified equation based on thermionic emission theory for the evaluation of the total effective activation energy. Note that previous published works promote this model for determining the total effective activation energy.[4,5]

$$J_{TE}(T) = A^* T^2 \exp \frac{-q\phi_{eSBH}}{k_B T} \tag{1}$$

where J_{TE} is the measured current density through the device, A^* is the effective Richardson constant, T is the corresponding temperature and $(q)\phi_{eSBH}$ is the total effective activation energy, which is interpreted as the effective barrier height. Without knowing the exact value of A^* the total effective activation energy can be extracted by measuring the I/V-characteristic at different temperatures and applying the natural logarithm to extract the barrier height of the previous equation. Equation 2 shows the then obtained expression.

$$\ln \frac{J_{TE}}{T^2} = \ln A^* - \frac{q\phi_{eSBH}}{k_B T} \tag{2}$$

Thus, by plotting $\ln \frac{J_{TE}}{T^2}$ (y-axis) as a function of $1000/T$ (x-axis), a so-called Richardson plot is obtained. Using the linear equation and setting the factors of the above equation correspondingly to y = kx + d, the individual parameters can be extracted. In the linear equation, d depicts the (natural logarithm) effective Richardson constant A^*. Hence, enabling to determine this parameter by evaluating the cross-point on the y-axis. Due to the simplified model used in the scope of this work, the extraction of A^* is not possible, as other unknown factors as the potential between the metal and semiconductor as well as the electron mass, are neglected. By analyzing the slope k the corresponding $q\phi_{eSBH}$ can be determined for a specific drain bias voltage V_D as depicted in Equation 3.

$$q\phi_{eSBH} = -k * k_B * 1000 \tag{3}$$

where k is the evaluated slope of the Richardson plot. Finally, $q\phi_{eSBH}$ can be plotted over V_D for the evaluation of the total effective activation energy. Extrapolation of the data points to $V_D = 0\,V$ is used to perform a careful estimation of the activation energy then, including initial effects of barrier lowering and tunneling at the tip of the barrier.

References

(1) Voort, G. F.; Asensio-Lozano, J. The Al-Si Phase Diagram. *Microscopy and Microanalysis* **2009**, *15*, 60–61.

(2) Durkan, C.; Welland, M. E. Size Effects in the Electrical Resistivity of polycrystalline Nanowires. *Physical Review B* **2000**, *61*, 14215–14218.

(3) Rhoderick, E.; Williams, R. In *Metal-Semiconductor Contacts*, 2nd ed.; Hammond, P., Grimsdale, R., Eds.; Oxford University Press: Oxford, 1988; p 268.

(4) Thanailakis, A.; Chan, D. S.; Northrop, D. C. Activation Energy for the slow States in the Aluminium-Germanium Contact. *Journal of Physics D: Applied Physics* **1972**, *5*, 325.

(5) Pitale, S.; Ghosh, M.; Singh, S.; Manasawala, H.; Patra, G.; Sen, S. Characteristics of Al/Ge Schottky and ohmic Contacts at low Temperatures. *Materials Science in Semiconductor Processing* **2021**, *130*, 105820.
